# A Non-Source Optical Fiber Sensor for Multi-Point Methane Detection

**DOI:** 10.3390/s24155031

**Published:** 2024-08-03

**Authors:** Li Ma, Xu Liu, Ganshang Si

**Affiliations:** 1 School of Civil and Hydraulic Engineering, Bengbu University, Bengbu 233030, China; 2 Hefei Institutes of Physical Science, Chinese Academy of Sciences, Hefei 230031, China; 3 School of Electronic and Electrical Engineering, Bengbu University, Bengbu 233030, China; 4 School of Earth and Space Sciences, University of Science and Technology of China, Hefei 230031, China; 5 Hefei Institute of Technology Innovation Engineering, CAS, Hefei 230031, China; 6 National Engineering Research Center of Coal Mine Water Hazard Controlling, Suzhou University, Suzhou 234000, China

**Keywords:** methane, TDLAS, optical fiber type, multi-point gas detection

## Abstract

Fast, accurate, real-time measurement of gas concentration is an important task for preventing coal mining disasters. In order to develop an accurate monitoring method for methane gas concentration at different locations in a mine environment, a non-source optical fiber sensor for multi-point methane detection has been developed in this paper. A 16-channel fiber splitter and a multi-channel time-sharing acquisition module are employed within the sensor, enabling simultaneous detection of methane gas at 16 points by a host. Furthermore, the methane sensors are connected to the monitoring host via an all-optical method, achieving non-source and long-range detection of methane. To assess the performance of the methane gas sensor, experiments were conducted to evaluate its detection range, response time, and stability. The results indicated that the average detection error was approximately 1.84% across the full range, and the response time did not exceed 10 s. The minimum detection limit of the sensor, as determined by the 1σ criteria, was obtained as 58.42 ppm. Additionally, the concentrations of methane gas measured at varying distances (1 km, 2 km, 5 km) were found to be essentially consistent over an extended period. These results suggest that the development of this non-source optical fiber sensor holds significant potential for providing a method for mine environment, multi-point online methane gas detection.

## 1. Introduction

With the development of the economy and society, the demand for energy increases accordingly. The power structure is mainly based on thermal power, which generates nearly 70% in China [1]. Due to the complex environment, safety accidents are very likely to occur during coal mining. Gas accidents account for the vast majority of coal mine accidents, causing significant casualties and economic losses, and becoming one of the major problems plaguing coal mine safety production [2]. Methane gas is often released during coal mining, and explosions are likely to occur when the methane concentration exceeds 5% (explosion limit 5–15%) [3]. Therefore, there is an urgent need to develop a method for on-site, accurate, and real-time monitoring of methane gas concentration.

At present, methane gas detection includes various technologies, such as photoacoustic spectroscopy (PAS), tunable semiconductor laser absorption spectroscopy (TDLAS), cavity ring-down spectroscopy (CRDS), Fourier transform infrared spectroscopy (FTIR), and so on [4,5,6,7,8]. Among the above technologies, TDLAS is favored by many researchers and is one of the effective methods for online monitoring of methane gas. Due to its advantages with high measurement accuracy, no cross-interference from other gases, and no need for frequent calibration in methane detection, many researchers have carried out a lot of research. Qiu X et al. developed an early-detection system for coal spontaneous combustion by laser dual-species sensor of CO and CH_4_ based on TDLAS, with a distributed-feedback diode laser emitting at 2.33 μm as a sensing light source for dual species [9]. The detection sensitivity for CH_4_ is about 0.20 ppm with a 2.5 s sampling time. To effectively monitor gas explosion and coal spontaneous combustion in coal mines, Li Y et al. developed a near-infrared wide-range dual-gas sensor system for simultaneous detection of methane and carbon monoxide in coal mine environments [10]. Based on the Allan deviation analysis, the limit of detection (LoD) of CH_4_ is 0.19 ppmv at an averaging time of 0.5 s. Zhang Q et al. designed a highly sensitive and reliable optical fiber TDLAS gas detection system for methane in situ monitoring in near space [11]. In response to the need for methane detection, researchers have developed a variety of detection methods based on TDLAS technology. However, the above methods can only detect methane at a single point and cannot achieve simultaneous detection at different locations.

In response to the needs of multi-point methane gas detection, Li Y et al. developed a quasi-distributed multipoint laser methane detection system based on TDLAS [12], a Vertical Cavity Surface Emitting Laser (VCSEL) has been designed, and its performance discussed. The system can realize online methane monitoring across 14 channels of sensors, with the maximum transmission distance for each sensor being 10 km; the measurement error was determined to be less than ±0.03% (of the gas concentration) when the methane volume concentration lies in the range up to 10% methane. To investigate natural gas fugitive leaks, Yang S et al. developed a Remote Methane Leak Detector (RMLD)–Unmanned Aerial Vehicle (UAV) system [13]. The system is composed of three major technologies: miniaturized RMLD (mini-RMLD) based on TDLAS, an autonomous quadrotor UAV, and simplified quantification and localization algorithms. The quantification algorithm from the research tended to underestimate the gas leak rates and yielded unreliable estimations in detecting leaks under 7 × 10^−6^ m^3^/s. Yang J et al. proposed a method for multipoint gas sensing by using intrapulse absorption spectroscopy and cascaded, branching gas cells [14]. A distributed feedback laser diode with pulsed current modulation over the threshold was used at 1650.9 nm. One full absorption peak of methane was scanned using 100 ns pulses, and the methane concentration in three 10 cm long gas chambers within a distance of 4 km was simultaneously measured. A noise equivalent concentration of approximately 10.96 ppm was achieved at 250 averages.

Our team has been conducting research on methane gas detection based on TDLAS for a long time and has achieved a series of results [15,16,17]. In 2016, team members conducted research on open natural gas leak methane gas detection technology, which can effectively detect CH_4_, C_2_H_4_, and C_2_H_2_ in the open environment within a response time of 2 s [18]. In 2020, team members developed a sensitive and selective dual-gas sensor system that combines multi-channel cells and time-sharing scanning assisted WMS technology for simultaneous detection of CH_4_ and C_2_H_2_, and the minimum detection limit for CH_4_ is 0.1 ppm [19].

On the basis of the above research, in order to further meet the demand for multi-point accurate and continuous detection of methane gas in coal mine environments, this paper developed a non-source optical fiber methane multi-point detection sensor based on TDLAS technology. A 16-channel fiber splitter and multi-channel time-sharing acquisition module are used in the sensor; methane gas at 16 points can be detected simultaneously by a host. Furthermore, the methane sensors and the monitoring host are connected by an all-optical method; it can achieve non-source and long-range detection of methane.

## 2. Methodology

### 2.1. TDLAS Theory

**Figure 1:**
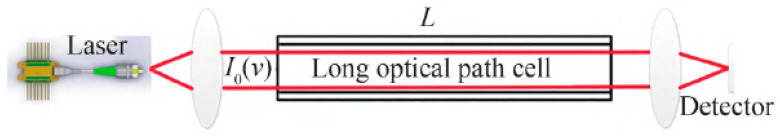
Gas measurement principle of TDLAS.

The TDLAS technique is derived from the Lambert–Beer Law [20,21], the law that describes the absorbance of the gas at a certain wavelength; it is proportional to the gas concentration, as shown in Figure 1. The relationship between transmission light intensity $I_{t}$ and incident light intensity $I_{0}$ is given by

(1) $$I_{t}=I_{0}\exp[-S(T)CPL\phi (\nu )]$$

where *ν* is wave number of incident light, *C* is the volume fraction of the gas, *P* and *T* are pressure and temperature, *φ*(*ν*) is the absorbing linear function after normalization of the integrated area, and *S*(*T*) is the intensity of the spectral line corresponding to the absorption spectrum when the gas temperature is *T*.

The relationship between concentration and absorbance can be derived from Equation (1); it can be expressed as:

(2) $$C=\frac{-In(I_{t}/I_{0})}{S(T)P\phi (\nu )L}=\frac{\alpha (\nu )L}{S(T)P\phi (\nu )L}$$

where *α*(*ν*) is the absorption coefficient of gas molecule at a specific frequency.

### 2.2. Wavelength Modulation and Harmonic Detection Theory

**Figure 2:**
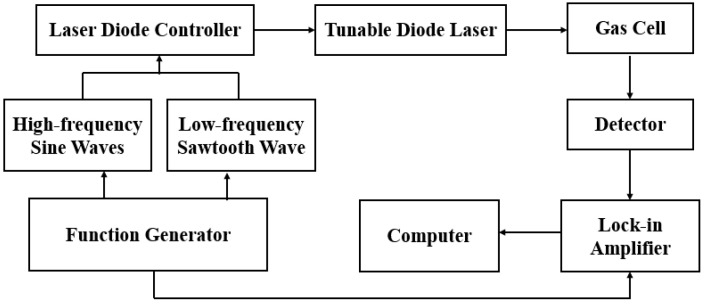
The schematic diagram of gas detection method.

Since the measurement of weak signals is easily affected by various noises, these noises generally have a greater impact when measuring at low frequencies, and the intensity of these noises is inversely proportional to the frequency, that is, the lower the frequency, the greater the noise intensity, and the impact of noise will decrease rapidly as the frequency increases. The emergence of modulation spectroscopy technology has largely solved the noise problem. First, the laser is modulated with a high-frequency signal, and then demodulated through a phase-locked amplifier to obtain a high-order harmonic signal that is positively correlated with the concentration of the gas to be measured. The gas detection method is shown in Figure 2.

In TDLAS wavelength modulation technology, a low-frequency sawtooth wave signal and a high-frequency sine wave signal are superimposed and input into the laser controller. The low-frequency sawtooth wave signal tunes the output wavelength of the laser by changing the injection current of the laser, so that it scans back and forth near the absorption peak. The high-frequency sine wave signal is used to modulate the laser at high frequency, thereby suppressing low-frequency noise.

On the basis of laser current, a sinusoidal modulation signal has been added. For a scan period, the incident laser wavelength ω can be given by [22,23]:

(3) $$\omega =\omega_{L}+\delta_{\omega }\cos\left(\omega_{m}t\right)$$

where $\omega_{L}$ is the wavelength of the center laser under modulation, δω is the modulation amplitude of frequency, and $\omega_{m}$ is the angular frequency of sinusoidal modulation. After the laser beam passes through the sample absorption cell, the intensity $I(\omega_{L})$ can be expressed as follows:

(4) $$I\left(\omega_{L},t\right)=\sum_{n=0}^{\infty }A_{n}\omega_{L}\cos\left(n\omega_{m}t\right)$$

Each harmonic component of $I\left(\omega_{L}\right)$ can be measured by phase-locked amplification.

(5) $$A_{n}\left(\omega_{L}\right)=\frac{2}{\pi }\int_{0}^{\pi }I_{0}\left(\omega_{L}+\delta_{\omega }\cos\theta \right)\exp\left[-\sigma \left(\omega_{L}+\delta_{\omega }\cos\theta \right)LC\right]\cos\left(n\theta \right)d\theta$$

(6) $$\theta =\omega_{m}t$$

For weakly absorbing gases, σLC≪1, Equation (5) can be expressed as:

(7) $$A_{n}\left(\omega_{L}\right)=\frac{2I_{0}CL}{\pi }\int_{0}^{\pi }-\sigma \left(\omega_{L}+\delta_{\omega }\cos\theta \right)LC\cos\left(n\theta \right)d\theta$$

A Taylor series expansion of *σ*(*ω*) in Equation (7), $A_{n}\left(\omega_{L}\right)$ can be expressed as:

(8) $$A_{n}\left(\omega_{L}\right)=\frac{2^{1-n}I_{0}CL}{n!}\delta \omega^{n}\frac{d^{n}\sigma }{d\omega^{n}}{|}_{\omega =\omega_{L}}$$

It can be seen from the above formula that the harmonic components at all levels are positively correlated with the concentration of the gas sample, and as the order of the harmonic component increases, the filtering ability for low-frequency noise should also be stronger. However, from Formula (8), it can be seen that as the harmonic order increases, the amplitude of the harmonic signal will drop rapidly, so when selecting harmonic components, the harmonic order should not be too high. Therefore, the second harmonic component is more suitable for characterizing the concentration measurement results of the gas sample and is suitable for the measurement of gas concentration.

(9) $$A_{2}\left(\omega_{L}\right)=\frac{I_{0}CL}{4}\delta \omega^{2}\frac{d^{2}\sigma }{d\omega^{2}}{|}_{\omega =\omega_{L}}$$

### 2.3. Experimental Setup Design

#### 2.3.1. Multi-Channal Optical Fiber Methane Gas Sensor

**Figure 3:**
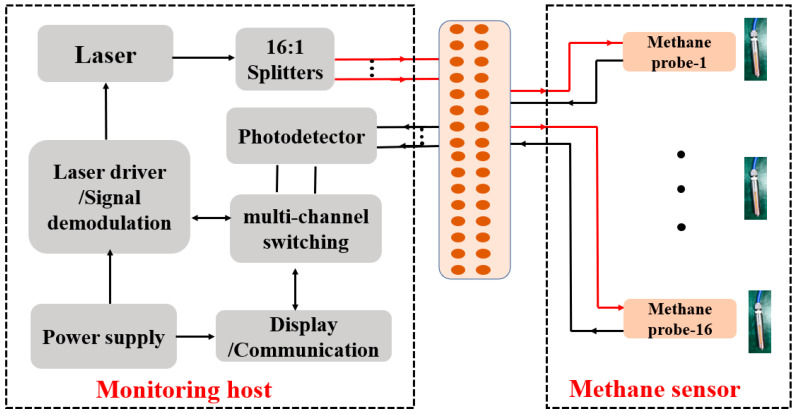
The schematic diagram of the sensor.

The sensor mainly consists of a monitoring mainframe and a methane sensing module, which are connected by optical fiber. The monitoring mainframe consists of a light source driving and signal acquisition and demodulation module, 16:1 optical beam splitter, photoelectric detection module, multiplex switching module, power supply module, display, and communication module. The methane sensing probe is a non-source sensor composed of pure optical components, and one monitoring host can be connected with up to 16 methane sensing probes. The design framework is shown in Figure 3.

A distributed feedback semiconductor laser is used in the sensor with a central wavelength of 1653.7 nm; the driving current is 90 mA, and the output power is 16.1 mW. The laser control parameters include current and temperature. The laser temperature is controlled at around 28.5 °C by TEC. The laser is converted into a 16-way beam by a 16:1 optical splitter and transmitted by optical fiber to the methane probe. The light beam absorbed by the methane gas is recoupled to the methane probe and transmitted to the photoelectric detector module by optical fiber. The photoelectric detector module consists of 16 photoelectric detectors, which convert the light signal into an electrical signal. Further, the electrical signal enters the multi-channel switching module through pre-amplification and enters the microcontroller processor for concentration inversion through time-sharing logic. Next, the concentration of methane gas at 16 different locations is obtained in turn. Finally, the information on the methane gas concentration at different locations is displayed on the human–machine interface.

#### 2.3.2. Methane Probe

**Figure 4:**
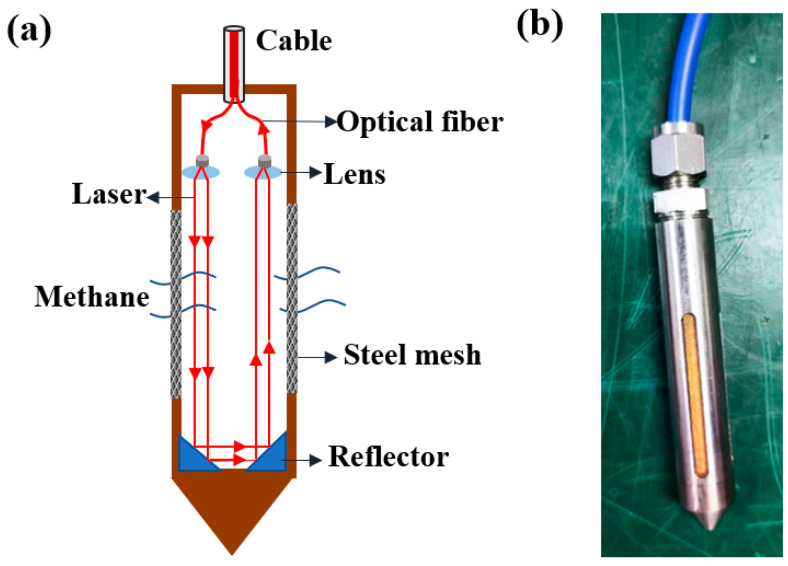
Methane probe design. (**a**) Schematic diagram; (**b**) physical diagram.

The non-source multi-channel optical fiber sensor is composed of 16 methane probes, which work independently, and the placement position is based on actual need. The diameter and length of the methane probe are 4 mm and 60 mm, respectively. The probe is mainly composed of armored fiber, lens, reflector, etc. The schematic and physical diagram of methane probe are shown in Figure 4.

As can be seen from Figure 4a, the laser beam is transmitted to the methane probe by an armored fiber, collimated by a lens, and reflected by two reflectors. The sensor and the test host are connected through armored optical cables. Each optical cable is encapsulated with 2-core single-mode optical fibers @ 1653 nm. The core diameter of the optical fiber is about 9 um, the numerical aperture is 0.13, and the optical fiber port is designed. The metallurgical powder filter used for sensor packaging is made of metal titanium and titanium alloy powder sintered and pressed at high temperature, with a pore size of 0.2–50 μm. This sensor uses a pore size of 5–10 um and a thickness of 2 mm, which has good permeability to gas molecules but can prevent water from entering. Of course, water vapor molecules can also enter, but water molecules have no absorption in this (1653.72 nm) band, so it has no effect on methane detection. The detector model used in this system is Hamamatsu (HAMAMATSU) G12936-32 (Shizuoka, Japan), which is installed on the demodulation host. The optical absorption path of the methane probe is approximately 10 cm, and all components are glued inside a stainless-steel housing, making it resistant to vibration and shock. The side of the sensing probe has a waterproof and breathable metallurgical powder filter, which effectively prevents dust and water from entering the gas chamber and polluting the lens, improving the robustness of the probe for applications in harsh environments.

## 3. Results and Discussion

To test the performance of the methane sensor, experiments are conducted using the full range of methane gases with different concentrations (0, 0.5%, 2.0%, 8.5%, 20.0%, 35.0%, and 70%). The performance of the system was further analyzed, including raw signal, correction error, minimum detection limit, response time, and stability. During detection, the driving current is triangular wave modulated output with a current range of 70–110 mA and a frequency of 10 Hz, and a 10 KHz high-frequency sine modulation signal is superimposed. The switching of 16 methane detection channels is achieved by the analog switch in the control system.

### 3.1. Methane Concentration Measurement in Full Range

**Figure 5:**
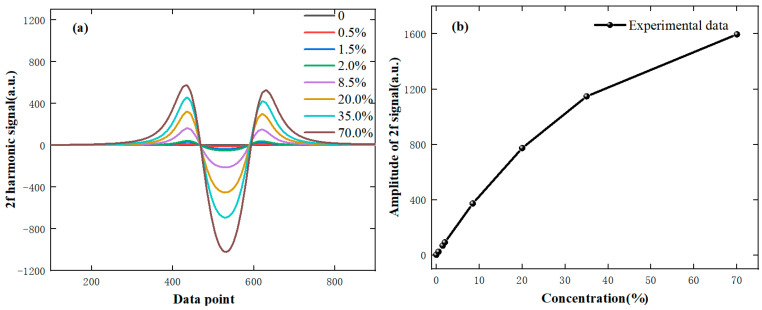
The measured 2f harmonic signal (**a**) and the relationship between concentration and 2f signal amplitude (**b**).

**Figure 6:**
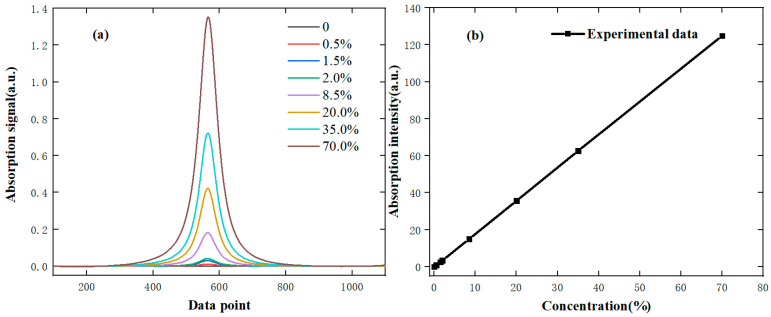
The measured absorption signals (**a**) and the relationship between concentration and absorption intensity (**b**).

In the laboratory environment, experiments are conducted with eight standard concentrations of methane gas (national secondary standards). The measurement results of the probes are analyzed, and the 2f signals at different concentrations are shown in Figure 5a. According to the theoretical part, it is known that the second harmonic amplitude is related to the laser intensity and the methane concentration. The relationship between the corrected second harmonic amplitude and the methane gas concentration is shown in Figure 5b. From the results, it shows that the linearity is good when the gas concentration is low, but the relationship is nonlinear in the full range. Therefore, the method of direct absorption spectroscopy is used in the high concentration range; the direct absorb signal of methane gas is shown in Figure 6.

The fitted direct absorption signal of methane gas is shown in Figure 6a, and the relationship between signal integration area and methane gas concentration is shown in Figure 6b. Clearly, it can be found that the linear relationship between concentration and absorption intensity is better. However, due to the limitation of absorbance path length and noise, the concentration inversion error is large when detecting low-concentration gases.

From the above results, when the gas concentration is low (≤2.0%), the linear relationship between the second harmonic signal amplitude and the concentration is good. As the methane gas concentration increases, there is no corresponding linear relationship. For direct absorption spectroscopy, the linear relationship is good when the gas concentration is higher. Therefore, this methane sensor employs a method that integrates direct absorption and wavelength modulation to achieve full-range and high-precision detection of methane gas.

### 3.2. The Performances of the Methane Sensor

**Figure 7:**
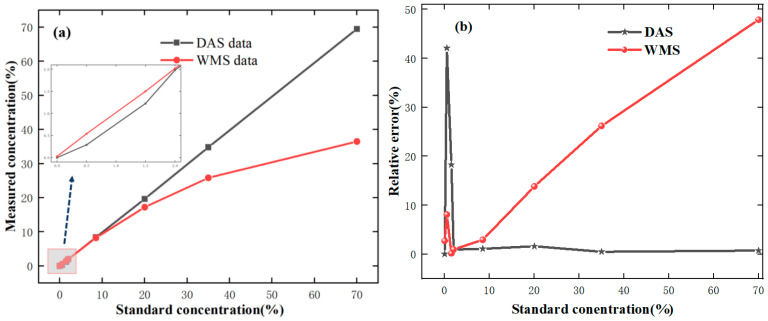
The correlation curves (**a**) and the relative errors (**b**) between the measured concentrations and the standard concentrations.

In order to verify the accuracy of DAS and WMS in full range, the relationship between the measured and the standard value of the different methods is presented in Figure 7a, and the relative errors are shown in Figure 7b.

For the WMS method, the linear relationship between the measured and standard values is good when the standard gas concentration is not greater than 2.0%, the concentration inversion error is small, and with the gas concentration increases, the error becomes large. For the DAS method, when the standard gas concentration is greater than 2.0%, the linear relationship is poor. When the gas concentration is small, the error is larger due to the limitation of absorption range and noise.

The above results show that the relative error is low when the gas concentration is small (≤2.0%) using the WMS method, and the error is low when the gas concentration is large (>2.0%) using the DAS method. Therefore, the measurement accuracy will be improved by combining the WMS and DAS methods; the results are shown in Table 1.

**Figure 8:**
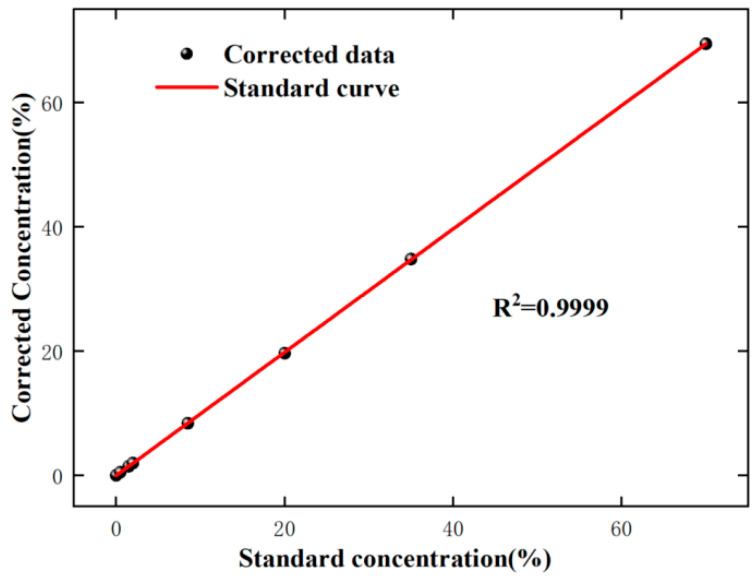
Relationship between gas corrected concentration and standard concentration.

The average measurement error of the DAS method for methane gas with eight different concentrations is 8.22%, and compared to low concentrations, the error is smaller for high-concentration gases. For the WAS method, the average measurement error is 12.69%, and the error is smaller for low-concentration gases compared to high concentrations. Based on the above analysis, taking advantage of the WMS and DAS, the average measurement error is only 1.84% in the full range. The relationship between the corrected concentration and the standard concentration is shown in Figure 8; it can be seen that the linear relationship is good (R^2^ = 0.9999).

**Figure 9:**
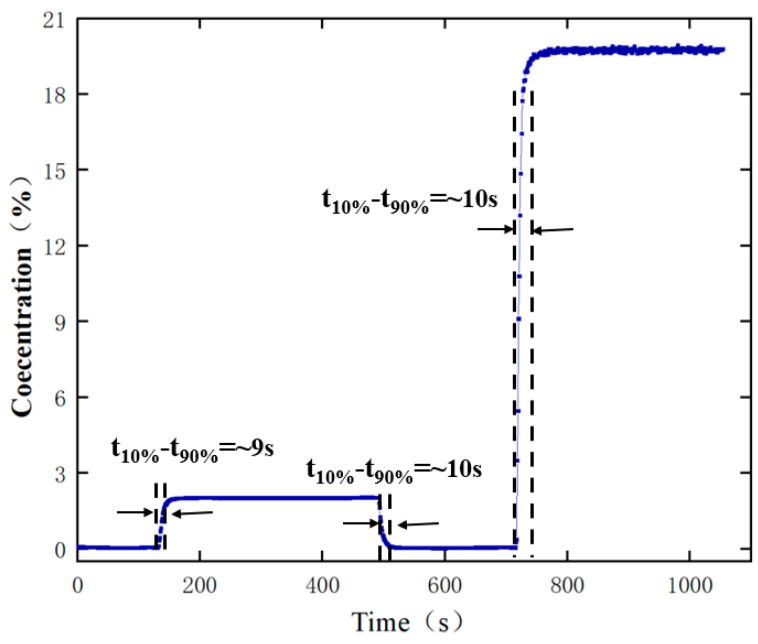
Response time test of the methane sensor.

In order to verify the response time of the sensor, methane gas at concentrations of 2.0% and 20.0% is measured by the experimental setup for a long time, and the relationship between gas concentration and measurement time is shown in Figure 9.

As shown in Figure 9, the rise time (10–90%) and the fall time (10–90%) are ~9 s and ~10 s (gas concentration in 0–2.0%), respectively. In addition, the rise time (10–90%) is about 10 s (gas concentration in 0–20.0%). The above results show that the sensor has a short response time and meets the demand for continuous methane gas detection.

**Figure 10:**
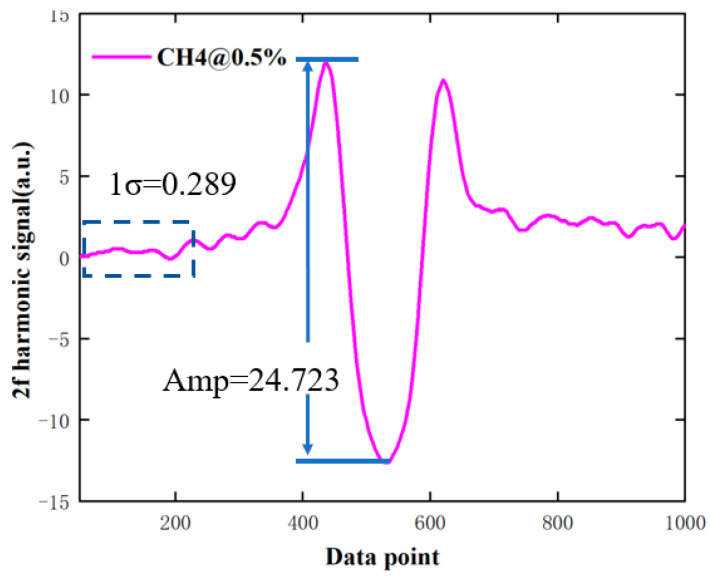
2f harmonic signal of CH_4_ at a concentration of 0.5%.

In fact, WMS can transfer the detection of absorption spectrum to the high frequency with better signal-to-noise ratio (SNR), so as to achieve the purpose of noise suppression. However, the background noise of the optical path overlaps with the harmonic signal, resulting in irregular fluctuations in the non-absorbing sidebands. This can be seen in Figure 10; the wing tilt of the harmonic signal of CH_4_ with a concentration of 0.5% indicates the presence of residual amplitude modulation.

The above research shows that the amplitude of 2f harmonic signal is 24.732, and the 1σ of the non-absorption wing is 0.289. According to the calculated SNR of 85.58, the minimum detection limit of the methane sensor can be inferred to be 58.42 ppm (1σ). In a laboratory environment, due to the optical path length of the probe being approximately 0.1 m, a minimum detectable column density 58.42 ppm × 0.1 m = 5.84 ppm m is obtained.

### 3.3. Methane Detection at Different Locations

**Figure 11:**
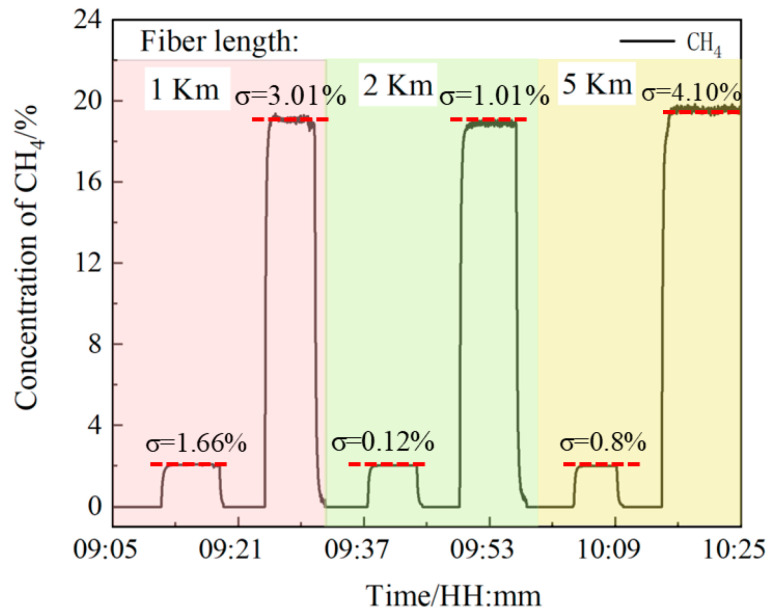
Methane gas measurement at different positions.

The detection distance may affect the laser light intensity and phase, which affects the accuracy of the measurement results. To verify that the sensor can make accurate measurements of methane gas at different locations, three different lengths optical fiber (1 km, 2 km, 5 km) are used to measure the methane gas (2.0% and 20.0%) for a long time; the results are shown in Figure 11.

It can be seen that the measurement results of methane gas concentration at three different distances are basically consistent. The relative standard deviation (RSD) of measurement concentrations for a long time is used to measure the stability of the sensor. The results show that the minimum and maximum values are 0.8% and 4.10%. The above analysis shows that the measurement results are almost independent of the detection distance, and the system performs well in terms of accuracy, stability, and responsiveness, meeting the demand for accurate multi-point methane gas detection.

## 4. Conclusions

In order to meet the continuity and accuracy requirements of multi-point methane gas detection, a non-source optical fiber sensor for multi-point methane detection is developed in this paper. A series of experiments are conducted to verify the performance of the sensor. The experimental results showed that the average measurement error of methane gas in the full range is 1.86% by combing DAS and WMS; it has a good linearity between standard concentration and corrected concentration with a correlation coefficient R^2^ = 0.9999. Furthermore, the response time is not more than 10 s. According to the 1σ criteria, the minimum detection limit of the sensor can be obtained as 58.42 ppm. At the same time, the experiment verified that different distance probes have almost no effect on the methane concentration measurement results. The above results indicate that the designed methane sensor meets the needs of continuous, accurate, and multi-point detection of methane gas and will play an important role in urban pipe galleries, coal mine warning, environmental safety monitoring, and other occasions. Although the sensor exhibits strong sensitivity and stability in testing, we acknowledge that specific challenges remain in practical engineering applications, especially concerning long-term stability. In the next phase, we will address these challenges by integrating advanced wavelength locking technology for modulation [24] and employing high-precision laser temperature control [25] to enhance the stability of the measurement results.

## Figures and Tables

**Table 1:** The relative error of concentration inversion by different methods.

Standard Concentration (%)	DAS			WMS			DAS/WMS	
	Measurement Concentration (%)	Error (%)	AVG (%)	Measurement Concentration (%)	Error (%)	AVG (%)	Error (%)	AVG (%)
0	0	0	8.22	0.02	2.0	12.69	2.0	1.84
0.5	0.29	42.08		0.54	8.07		8.07	
1.5	1.22	18.67		1.50	0.14		0.14	
2.0	1.98	1.00		2.01	0.5		0.5	
8.5	8.40	1.18		8.25	2.94		1.18	
20	19.68	1.61		17.23	13.85		1.61	
35	34.83	0.49		25.84	26.17		0.49	
70	69.49	0.73		36.50	47.86		0.73	

## Data Availability

The data presented in this study are available on request from the corresponding author.
